# MultiV_Nm: a prediction method for 2′-O-methylation sites based on multi-view features

**DOI:** 10.3389/fgene.2025.1608490

**Published:** 2025-05-27

**Authors:** Lei Bai, Fei Liu, Yile Wang, Junle Su, Lian Liu

**Affiliations:** ^1^ School of Physics and Opto-Electronic Technology, Baoji University of Arts and Sciences, Baoji, China; ^2^ School of Computer Sciences, Shannxi Normal University, Xi’an, China

**Keywords:** 2′-O-methylation sites, multi-view, convolutional neural networks, graph attention network, cross attention mechanism

## Abstract

As a crucial class of chemical modifications, 2′-O-methylation modification (abbreviated as Nm) is widely distributed in various organisms and plays a very important role in normal cellular physiological activities and the occurrence and development of diseases. Accurate prediction of Nm modification sites can provide important references for the diagnosis and treatment of diseases, as well as identifying for potential drug targets. Aiming at the current problems of unstable performance caused by the use of single features and the need to improve the prediction accuracy of Nm modification sites, this paper proposes MultiV_Nm, a prediction method for Nm sites based on multi-view features. MultiV_Nm extracts the features of Nm sites from multiple dimensions, including sequence features, chemical characteristics, and secondary structure features. By integrating the powerful local feature extraction ability of convolutional neural networks, the ability of graph attention networks to capture global structural information, and the efficient interaction advantage of cross-attention mechanisms for different features, it deeply explores and integrates multi-view features, and finally realizes the prediction of Nm modification sites. The results of cross-validation and independent tests show that this method exhibits significant advantages in key evaluation indicators such as precision, recall, and accuracy, and can effectively improve Nm sites prediction performance. The proposal of MultiV_Nm not only provides a powerful tool for the study of Nm modification but also offers new ideas for predicting other RNA modification sites.

## 1 Introduction

In recent years, with the in-depth research in the field of epitranscriptomics, RNA modifications, as a crucial epigenetic mechanism for gene expression regulation, have attracted extensive attention in the field of biomedical research (Boccaletto et al., 2018). Different from DNA and histone modifications, RNA modifications regulate various aspects of RNA metabolism through dynamic and reversible chemical modification methods, have a significant impact on biological processes such as individual development and disease occurrence (Roundtree et al., 2017), and are closely related to the occurrence and development of a variety of diseases (Li et al., 2018; Cui et al., 2022). Currently, more than 200 different types of RNA chemical modifications have been identified in eukaryotes (Boccaletto et al., 2022). Among them, 2′-O-methylation (abbreviated as Nm) is an extremely important and widely existing type of RNA modification. Catalyzed by 2′-O-methyltransferases, it adds a methyl group to the 2′-hydroxyl group of RNA (Nicolai et al., 2016). Nm exists in the 2′-hydroxyl ribose moieties of all four ribonucleosides (Xuan et al., 2018), namely, 2′-O-methylcytidine (Cm), 2′-O-methyladenosine (Am), 2′-O-methylguanosine (Gm), and 2′-O-methyluridine (Um). Widely present in various RNA molecules (Li et al., 2024), this modification plays a key role in maintaining normal physiological functions in organisms (Yu et al., 2004). The distribution of Nm is extremely extensive, and it can be found in rRNA, mRNA, tRNA, snRNA, piRNA as well as human viruses (Wu et al., 2024). In rRNA, the enzyme fibrillarin (FBL) can catalyze the Nm reaction. In the breast cancer cell model, the mutation of the tumor suppressor gene TP53 will increase the expression of FBL, which in turn leads to an elevated level of Nm modification in rRNA and abnormal translation of the internal ribosome entry site (IRES) oncogenes (Marcel et al., 2013). In mRNA, Nm modifications occur both at the 5′ cap and internal sites. The modification of the 5′ cap can protect the mRNA and regulate immune recognition; the modifications at internal sites can affect the translation efficiency and mRNA stability, and are also associated with viral infections (Picard-Jean et al., 2018). The Nm modification can stabilize the L-shaped tertiary structure of tRNA, enhance its thermal stability, and contribute to its correct folding. Moreover, it will also affect the recognition process of the codon by the tRNA anticodon during translation (Agris et al., 2017). The dysregulation of Nm modification in snRNA may disrupt the splicing process, resulting in the generation of erroneous mRNA and protein sequences, and may potentially trigger diseases such as cancer and splicing-related genetic disorders (Blijlevens et al., 2021). The study by Lim et al. has demonstrated that the protein HENMT1 is a key regulator of Nm modification in mammalian piRNA (Lim et al., 2015). Moreover, the Nm modification of the genomes of RNA viruses such as human immunodeficiency virus type 1 (HIV-1) and West Nile virus (WNV) may help them evade the host’s innate immune response (Daffis et al., 2010; Ringeard et al., 2019), providing potential targets for antiviral treatment. In addition, a large number of studies have continuously shown that RNA Nm modification is closely related to a variety of human diseases, such as hepatocellular carcinoma and lung adenocarcinoma (Song et al., 2023).

To deeply explore the functional mechanism of Nm modification, researchers have developed a series of biological experimental techniques (Yu et al., 1997; Maden et al., 1995; Zhang et al., 2023). However, these traditional techniques generally suffer from the problems of long time consumption and high cost, and it is difficult to meet the needs of biological research for efficient and rapid detection. With the rapid development of sequencing technologies, nucleotide sequence data have shown explosive growth, which has opened up a new path for computational prediction methods. Predicting Nm modification sites through computation can effectively make up for the deficiencies of experimental techniques and provide strong support for relevant research.

Currently, the computational tools for predicting RNA Nm modification sites are relatively limited. Chen et al. (2016) constructed the first computational tool for identifying Nm modification sites by using support vector machine (SVM) for classification based on the encoding methods of nucleotide chemical properties and nucleotide composition features. However, this model was only constructed based on human data, and its prediction performance in other species has not been fully demonstrated. Qiu et al. (2017) incorporated the sequence coupling effect into the General Pseudo k-tuple Nucleotide Composition (General PseKNC) and used a variety of machine learning algorithms to construct an ensemble classifier. By combining and optimizing different algorithms, the prediction accuracy and stability were improved. In 2018, Yang et al. (2018) developed a sequence-based predictor iRNA-2OM for humans. By fusing chemical properties, nucleotide composition and PseKNC features, and combining feature selection methods with incremental feature selection (IFS), they obtained the optimal feature set and then constructed the prediction model. Zhou et al. (2019) developed the predictor NmSEER2.0 for Nm modification sites in the genomes of human HeLa and HEK293 T cells. This tool is based on random forest (RF) and multiple encoding schemes, and its AUC value reaches 0.862, showing good prediction performance. Ao et al. (2022) constructed the predictor NmRF based on the optimal mixed features and random forest classifier. By fusing nucleotide chemical properties, binary features and dinucleotide position-specific features, and then using a two-step strategy of combining the light gradient boosting algorithm with IFS feature selection, NmRF obtained the optimal feature set.

In addition, deep learning has gradually been applied to this field. The Deep-2′-O-Me method proposed by Mostavi et al. (2018) uses the dna2vec embedding method improved based on the word2vec model to learn the complex feature representations of pre-mRNA sequences, and fine-tunes them with the help of a convolutional neural network (CNN). In the test scenarios of both balanced and imbalanced datasets, the AUC and AUPRC scores both reach 0.9, significantly outperforming existing algorithms. iRNA-PseKNC (Tahir et al., 2019) uses PseKNC to extract the features of RNA sequences, and utilizes the feature learning ability of convolutional neural networks to automatically extract deep-level features and explore the complex relationships between RNA sequences and Nm sites. DeepOMe (Li et al., 2021) combines CNN and bidirectional long short-term memory networks (BLSTM), which enables it to accurately predict the Nm sites in the human transcriptome. Pichot et al. (Pichot et al., 2022) utilized the RiboMethSeq dataset and employed the random forest algorithm to construct a predictive model for analyzing Nm sites in RNA. This model was trained on a large number of human rRNA datasets with known modification profiles, and the modification profiles of other eukaryotic rRNAs (*Saccharomyces cerevisiae* and *Arabidopsis thaliana*) determined through experiments were used to evaluate the performance of the predictive model. For each type of Nm methylation, i2OM (Yang et al., 2023) combines one-way analysis of variance with mutual information to rank the sequence features, to obtain the optimal feature subset. Subsequently, four predictors based on eXtreme Gradient Boosting (XGBoost) or SVM are used to identify four types of Nm sites. BERT2OME (Soylu and Sefer, 2023) combines the BERT-based model with CNN to infer the relationship between the modification sites and the RNA sequence content. The results show that BERT2OME reduces the time consumed in biological experiments, and outperforms existing methods in terms of multiple metrics across different datasets and species. A large number of cutting-edge studies have shown that Nm modification is widely involved in key biological processes such as RNA splicing, transportation, and stability regulation. Accurately identifying Nm methylation modification sites helps deeply understand the pathogenesis of diseases and provides an important basis for developing new diagnostic and treatment strategies. However, current prediction methods for Nm methylation sites have obvious deficiencies. Most existing models rely only on single features, making it hard to comprehensively capture information in RNA sequences and structures, leaving significant room for improving the models’ prediction accuracy and stability.

To overcome this technical hurdle, we propose MultiV_Nm, an innovative prediction framework for Nm methylation sites using multi-view features. It extracts nucleotide sequence features through one-hot encoding, explores chemical properties and obtains RNA secondary structural features. The model combines convolutional neural networks for local feature extraction, graph attention networks for global relationship modeling, and a cross-attention mechanism for feature interaction. This integration enables in-depth understanding of multi-view features, significantly enhancing the prediction accuracy of Nm methylation sites.

## 2 Materials and methods

### 2.1 Datasets construction

To predict the Nm methylation modification sites, we utilized the Nm-seq technology to collect the information of Nm methylation modification sites at single-base resolution from two types of cells, namely, HeLa and HEK293T cells (see Table 1). During the research process, the Nm methylation sites detected in these two types of cells were defined as positive Nm methylation modification sites. To construct the negative sample set, we randomly selected an equal number of sites as the positive sites from the regions containing the positive samples. At the same time, we excluded the sites located in the ambiguous regions that could be mapped to multiple genes to ensure the accuracy and reliability of the data.

**TABLE 1:** Single-base resolution datasets in Nm prediction.

Id	Cell	Note	Technique	Source
1	HeLa	Control	Nm-seq	Dai et al. (2017)
2	HEK293 T	Control	Nm-seq	Dai et al. (2017)

According to the statistics, a total of 7,193 positive Nm methylation sites were finally collected. Among them, there were 1,591 Am type sites, accounting for 22.1% of all Nm sites; 1,471 Gm type sites, accounting for 20.5%; 1878 Cm type sites, accounting for 26.1%; and 2,253 Um type sites, accounting for 31.3%. To ensure the balance between positive and negative samples, the number of negative samples is the same as the positive. Subsequently, 3,696 samples were extracted from the collected samples as the test samples, including 1,848 positive samples and 1,848 negative samples. According to the proportion of each type of Nm in the total Nm, the numbers of Am, Gm, Cm, and Um in the test samples were 352, 300, 490, and 706, respectively, and the number of negative samples was the same as the positive. The remaining samples were used as the training samples, totaling 10,690, including 5,345 positive samples and 5,345 negative samples.

To obtain the sequence data for model training and testing, taking the Nm methylation sites as the center, we extended 25 base pairs (bp) upstream and downstream respectively, and finally obtained the Nm methylation modification site sequences with a length of 51 bp, laying a solid data foundation for the subsequent prediction analysis.

### 2.2 Methods

#### 2.2.1 Overall model architecture

**FIGURE 1:**
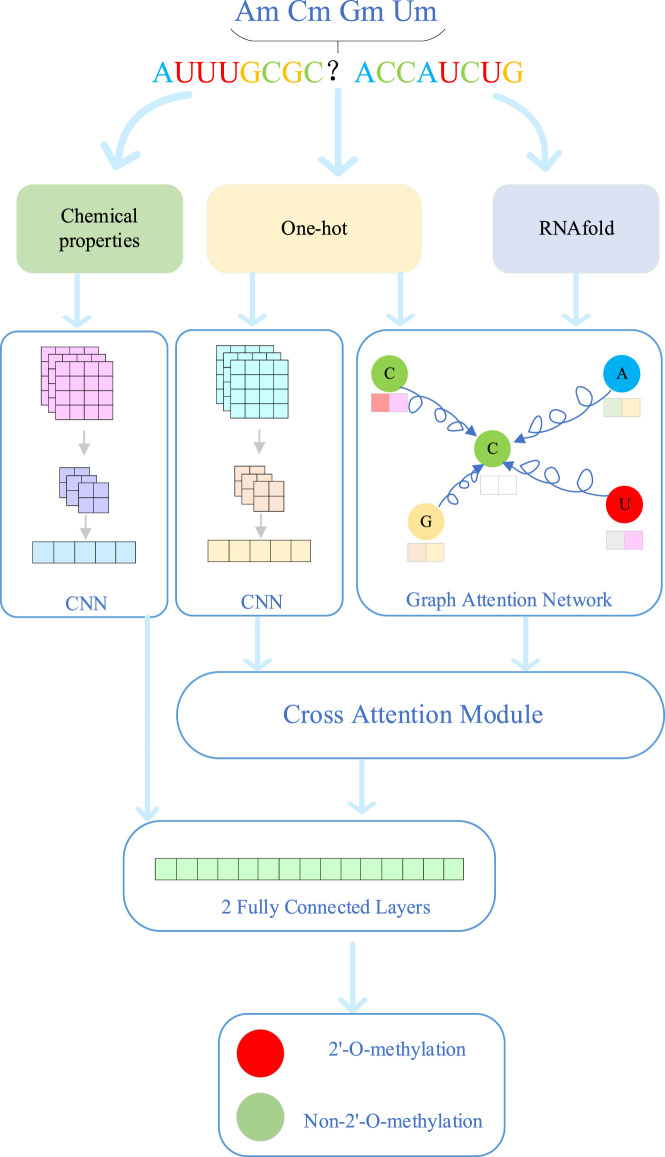
Detailed flowchart of MulitV_Nm.

The overall model architecture of MultiV_Nm is shown in Figure 1. First, for the known RNA sequences, the model extracts the features from sequence and chemical properties. For sequence features, one-hot encoding is used for feature extraction, which completely preserves the genetic and regulatory information contained in the arrangement order of nucleotides. At the same time, a quantitative analysis is carried out on the chemical property features of RNA molecules to explore their potential associations with modification sites. In addition, through the RNAfold software, the secondary structure features of RNA are analyzed to obtain the local folding information formed by base pairing of the molecules.

The prediction process of MultiV_Nm involves four modules: the convolutional neural network (CNN), the graph attention network (GAT), the cross-attention mechanism, and the fully connected layers. The CNN is mainly used to extract the deep features of sequences and chemical properties. The GAT extracts the deep features of the secondary structure through the spatial relationships and connection information between nodes. The cross-attention module fuses the sequence features and secondary structure features to achieve feature complementarity. The fully connected layers obtain the final prediction results by integrating the chemical property features and the complementary features. First, since the CNN has a powerful ability of automatic learning in local feature extraction, MultiV_Nm combines CNN with the pooling layer to deeply mine the sequence features and chemical property features extracted in the early stage. It captures the deep features hidden in the data and enhances the expression ability of sequence features and chemical property features. Second, the GAT provides strong support for the analysis of secondary structure features. The RNA secondary structure is composed of many nodes. By introducing the attention mechanism, GAT can make full use of the information of nodes and edges, accurately capture the interactions of nucleotides in the spatial structure, and explore the deep features of the secondary structure. Third, the sequence features mainly carry genetic information and regulatory information of biological processes, while the secondary structure features intuitively show the local folding morphology of RNA molecules. To give full play to the advantages of both, MultiV_Nm introduces a cross-attention module to fuse the deep sequence features and structural features. This module can automatically learn the relationships between the two types of features, adaptively adjust the fusion weights, and achieve efficient integration of features. Finally, the deep chemical property features are concatenated with the features fused by the cross-attention module. Through two fully connected layers, the integrated features are further analyzed and processed, and finally, the prediction results of Nm methylation modification sites are output.

#### 2.2.2 Feature representation

##### 2.2.2.1 Sequence feature representation

One-hot encoding is a technique for converting categorical variables into vector representations. For a categorical variable with *n* different categories, One-hot encoding represents each category as a vector of length *n*, in which only one element is 1 and the rest are all 0. The RNA sequence is composed of adenine (A), cytosine (C), guanine (G), and uracil (U), and its character set is {A, C, G, U}. We assign a unique integer index to each character in the character set and create a dictionary to map characters to indices. The dictionary is created as follows: {A: 0, C: 1, G: 2, U: 3}. In this dictionary, each base corresponds to a unique integer index, which facilitates subsequent encoding operations.

For each character, its corresponding index is obtained according to the encoding dictionary. Then, the value is set to 1 at the corresponding position in the feature vector. Suppose the index corresponding to the current character is *i*, then the *i*th element in the feature vector is set to 1. Therefore, A is encoded as [1, 0, 0, 0], C is encoded as [0, 1, 0, 0], G is encoded as [0, 0, 1, 0], and U is encoded as [0, 0, 0, 1]. For example, a sequence seq = [AACUG] can be encoded as the matrix shown in Equation 1. For a sequence with a length of 51 bp, it is encoded as a 51*4 matrix through one-hot encoding.

$$seq=\left[\begin{array}{cccc} 1 & 0 & 0 & 0\\ 1 & 0 & 0 & 0\\ 0 & 1 & 0 & 0\\ 0 & 0 & 0 & 1\\ 0 & 0 & 1 & 0 \end{array}\right]$$ (1)

##### 2.2.2.2 Chemical property features representation

Each nucleotide in RNA can be represented by three features according to its different chemical properties (Liu et al., 2020). C and U have only one ring structure, while A and G have two rings; both A and C contain an amino group, while both G and U contain a keto group; when forming the secondary structure, the hydrogen bonds between G and C are relatively strong, while the hydrogen bonds between A and U are relatively weak. Based on these three features, a nucleotide can be represented by a three-dimensional vector *S* = (*x*_*i*_, *y*_*i*_, *z*_*i*_, such as Equation 2):

$$x=\left\{\begin{array}{l} 1,s\in \left\{A,\left.G\right\}\right.\\ 0,s\in \left\{C,\left.U\right\}\right. \end{array}\right.,y=\left\{\begin{array}{l} 1,s\in \left\{A,\left.C\right\}\right.\\ 0,s\in \left\{G,\left.U\right\}\right. \end{array}\right.,z=\left\{\begin{array}{l} 1,s\in \left\{A,\left.U\right\}\right.\\ 0,s\in \left\{C,\left.G\right\}\right. \end{array}\right.$$ (2)

Therefore, A, C, G, and U can be encoded as [1, 1, 1], [0, 1, 0], [1, 0, 0], [0, 0, 1], and [0, 0, 1], respectively.

##### 2.2.2.3 Secondary structure features representation

To extract the secondary structure features of RNA sequences, RNAfold (Lorenz et al., 2011) in the ViennaRNA package (http://rna.tbi.univie.ac.at/cgi-bin/RNAWebSuite/RNAfold.cgi) was used to predict the RNA secondary structure. According to the given RNA sequence, RNAfold can predict the possible secondary structure of RNA by calculating thermodynamic parameters and return the results in the form of dot-bracket. In order to show the relationship between bases in the RNA sequence, we constructed a base-base relationship matrix. In the dot-bracket, a “dot” represents an unpaired base, and it is set to 0 in the base-base relationship matrix. The left parenthesis “ (“ and the right parenthesis”)” are used to represent paired bases. The left and right parentheses appear in pairs. The left parenthesis is placed at the position of one of the paired bases, and the right parenthesis is placed at the position of the other paired base. For example, if the *ith* base is paired with the *jth* base (i < j), then the *ith* position is represented by a left parenthesis and the *jth* position is represented by a right parenthesis, and then the element in the *ith* row and *jth* column as well as the element in the *jth* row and *ith* column of the base-base relationship matrix is set to 1. Therefore, for an RNA sequence with a length of 51, a secondary structure feature matrix *X*_*str*_ of 51*51 can be obtained.

#### 2.2.3 Feature learning

In order to learn the low-dimensional representation of Nm sites, we take the extracted sequence features and chemical property features as the inputs of the convolutional neural network (CNN) (Wang et al., 2024b) respectively. This model is composed of an input layer, a convolutional layer, a pooling layer, and a fully connected layer. $X_{seq}^{\left(1\right)}$ and $X_{chem}^{\left(1\right)}$ respectively represent the convolutional features obtained from the convolutional layer (such as Equation 3):

$$\begin{array}{l} X_{seq}^{\left(1\right)}=\text{ReLU}\left(W_{seq}⊗X_{seq}+b_{seq}\right)\\ X_{chem}^{\left(1\right)}=\text{ReLU}\left(W_{chem}⊗X_{chem}+b_{chem}\right) \end{array}$$ (3)

among them, $X_{seq}$ and $X_{chem}$ respectively represent the extracted sequence and physical and chemical property features. $W_{seq}$ and $W_{chem}$ represent the weight matrices of the convolutional kernels, $b_{seq}$ and $b_{chem}$ are the bias terms. ⊗ represents the convolution operation.

Then, the output of the convolutional layer goes through the pooling layer and the fully connected layer, and the finally obtained feature representation is as follows Equation 4:

$$\begin{array}{l} X_{seq}^{\left(2\right)}=f_{\text{fc}}\left(\left(f_{\text{Pool}}\left(f_{\text{CNN}}\left(X_{seq}\right)\right)\right)\right)\\ X_{chem}^{\left(2\right)}=f_{\text{fc}}\left(\left(f_{\text{Pool}}\left(f_{\text{CNN}}\left(X_{chem}\right)\right)\right)\right) \end{array}$$ (4)

where $X_{seq}^{\left(2\right)}$ represents the sequence feature representation extracted by the convolutional neural network module, and $X_{chem}^{\left(2\right)}$ represents the chemical property feature representation extracted by the convolutional neural network module.

For the secondary structure features, we use the Graph Attention Network (GAT) (Veličković et al., 2018) for feature extraction. The GAT is composed of multiple stacked graph attention layers. Each graph attention layer contains multiple attention heads. Each attention head independently calculates the feature representation of nodes, and then combines these representations (such as concatenation or averaging) to obtain richer node features. Compared with traditional graph neural networks, the GAT has higher flexibility and expressive power.

The input of the graph attention layer is $h=\left\{h_{1},h_{2},...,h_{N}\right\},h_{i}\in R^{F}$ , where *N* is the number of nodes and *F* is the number of features for each node. This layer generates a new set of node features $h^{\prime }=\left\{h_{1}^{\prime },h_{2}^{\prime },...,h_{N}^{\prime }\right\},h_{i}\in R^{F^{\prime }}$ as the output.

To obtain sufficient expressive power to transform the input features into higher - level features, at least one learnable linear transformation is required. For this purpose, as an initial step, a shared linear transformation with parameters $W\in R^{F^{\prime }\times F}$ is applied to each node. Then, self - attention of the nodes is performed, that is, a shared attention mechanism.

$$e_{ij}=\text{atten}\left(Wh_{i},Wh_{j}\right)$$ (5)

among them, *e*_*ij*_ represents the importance of the feature of node *j* to node *i*. The attention mechanism atten (·) is a single-layer feedforward neural network, with the parameter being $\vec{a}\in R^{2F^{\prime }}$ .

In order to make the attention weights easy to compare among different nodes, we use the softmax function to standardize the selections for all *j*:

$$\alpha_{ij}={\text{softmax}}_{j}\left(e_{ij}\right)=\frac{\exp\left(e_{ij}\right)}{\sum_{k\in N_{i}}\exp\left(e_{ik}\right)}$$ (6)

where *N*_*i*_ represents the neighborhood of node *i*.

Combining the above Equations 5, 6, the complete form of the attention mechanism can be written as:

$$\alpha_{ij}=\frac{\exp\left(\text{LeakyReLU}\left({\vec{a}}^{T}\left[Wh_{i}\|Wh_{j}\right]\right)\right)}{\sum_{k\in N_{i}}\exp\left(\text{LeakyReLU}\left({\vec{a}}^{T}\left[Wh_{i}\|Wh_{k}\right]\right)\right)}$$ (7)

among them, || represents the concatenation operation. Next combining Equation 7, the neighborhood representations of the nodes are linearly accumulated according to the attention weights to obtain the final output representation:

$$h_{i}^{\prime }=\sigma \left(\sum_{j\in N_{i}}\alpha_{ij}Wh_{j}\right)$$ (8)

To stabilize the learning process and enrich the feature representation, GAT usually adopts the multi-head attention mechanism. By using *K* independent attention heads for calculation and then averaging the outputs of these heads, by modifying Equation 8, the final node features are obtained:

$$h_{i}^{\prime }=\sigma \left(\frac{1}{K}\sum_{k=1}^{K}\sum_{j\in N_{i}}\alpha_{ij}^{k}W^{k}h_{j}\right)$$ (9)

where *K* is the number of attention mechanisms and *W*_*k*_ is the weight matrix for the *kth* attention mechanism.

Through the Graph Attention Network, we can obtain the deep representation $X_{str}^{\left(2\right)}$ of the secondary structure features of RNA through Equation 9.

#### 2.2.4 Cross-attention module

**FIGURE 2:**
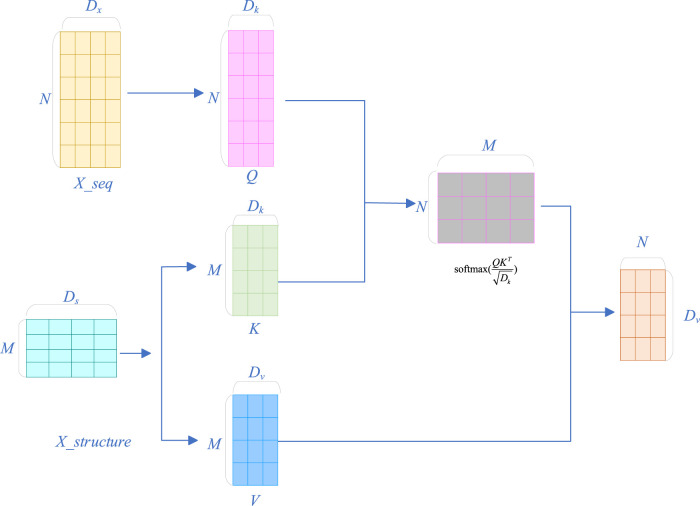
Flowchart of the cross-attention mechanism.

The cross-attention module is a special attention mechanism used to process two different types of features. In this study, these two features are the sequence feature $X_{seq}^{\left(2\right)}$ and the secondary structure feature $X_{str}^{\left(2\right)}$ respectively. The cross-attention mechanism allows the model to adaptively focus on the relevant information in the other feature when processing one feature, thereby achieving effective fusion of the two features. The specific process is shown in Figure 2.

First, linear transformations are performed on the deep representation of sequence features $X_{seq}^{\left(2\right)}$ and the deep representation of secondary structure features $X_{str}^{\left(2\right)}$ respectively to obtain the query vector, key vector, and value vector respectively.

$$\begin{array}{l} Q=X_{seq}^{\left(2\right)}W_{q}\\ K=X_{str}W_{k}\\ V=X_{str}W_{v} \end{array}$$ (10)

where $W_{q}\in R^{D_{x}\times D_{k}}$ , $W_{k}\in R^{D_{y}\times D_{k}}$ , and $W_{v}\in R^{D_{y}\times D_{v}}$ are learnable parameter matrices. $Q\in R^{N\times D_{k}}$ is the query vector matrix, $K\in R^{M\times D_{k}}$ is the key vector matrix, and $V\in R^{M\times D_{v}}$ is the value vector matrix.

Then, by calculating the similarity between the query vector and the key vector, the attention scores are obtained, and the scores are normalized to the interval [0, 1] through the softmax function:

$$A=\text{softmax}\left(\frac{QK^{T}}{\sqrt{D_{k}}}\right)$$ (11)

where $A=\in R^{N\times M}$ is the attention score matrix.

Then, combining Equations 10, 11, the value vectors are weighted and summed according to the attention scores to obtain the fused features.

O=AV (12)

where $O\in R^{N\times Dv}$ is the fused feature matrix.

We concatenate $X_{chem}^{\left(2\right)}$ with the feature matrix *O* fused by the cross - attention module, and then input the result into two fully - connected layers to obtain the final prediction result.

#### 2.2.5 Model training

We use binary cross entropy loss as the loss function, see Equation 13:

$$L=-\frac{1}{N}\sum_{i=1}^{N}\left[y_{i}\log\left({\hat{y}}_{i}\right)+\left(1-y_{i}\right)\log\left(1-{\hat{y}}_{i}\right)\right]$$ (13)

where *i* represents the *i*th Nm, and *y*_*i*_ represents the true label, ${\hat{y}}_{i}$ represents the probability that the model predicts the class as positive. To minimize the loss function, we use the Adam optimizer (Kingma and Ba, 2014) to minimize the loss function.

### 2.3 Evaluation metrics

To evaluate the performance of the model, 5-fold cross-validation is used to evaluate the performance of the model. We plotted the Receiver Operating Characteristic (ROC) curve and the Precision-Recall curve, and calculated the Area Under the Curve of the ROC (*AUC*) and the Area Under the Precision-Recall Curve (*AUPR*) to assess the model’s performance. The ROC curve is obtained by means of the True Positive Rate (*TPR*) and the False Positive Rate (*FPR*) at different scoring thresholds, and the Precision-Recall curve is obtained based on precision and recall at different scoring thresholds. The AUC is insensitive to whether the sample classes are balanced. In the case of highly imbalanced data, the performance is still overly ideal and cannot well reflect the actual situation. Under extremely imbalanced data (with fewer positive samples), the Precision-Recall (PR) curve may be more practical than the ROC curve. We used the AUC and AUPR as the main evaluation metrics. In addition, we adopted Accuracy (*ACC*), Matthews Correlation Coefficient (*MCC*), and *F1_score* to present the results of the model, which are defined as follows Equation 14:

$$\begin{array}{l} TPR=\frac{TP}{TP+FN}\\ FPR=\frac{FP}{FP+TN}\\ Precision=\frac{TP}{TP+FP}\\ Recall=\frac{TP}{TP+FN}\\ ACC=\frac{TP+TN}{TN+FP+TP+FN}\\ MCC=\frac{TP\times TN-FP\times FN}{\sqrt{\left(TP+FP\right)\left(TN+FN\right)\left(TP+FN\right)\left(TN+FP\right)}}\\ F1\_score=\frac{2\times Precision\times Recall}{Precision+Recall} \end{array}$$ (14)

where *TP* represents the number of positive samples that are predicted as positive samples; *FP* represents the number of negative samples that are predicted as positive samples; *TN* represents the number of positive samples that are predicted as negative samples; *FN* represents the number of negative samples that are predicted as negative samples.

## 3 Results and discussion

### 3.1 Adjustment of parameters

In the MultiV_Nm model, we made fixed settings for some key parameters. Specifically, when using the convolutional neural network to process different features, the number of channels is determined according to the dimension of the features. That is, when processing sequence features, the number of channels is set to 4; when processing chemical property features, the number of channels is set to 3. Meanwhile, the size of the convolutional kernel is uniformly set to 3. For the graph attention network, we set the number of attention heads to 8 and the size of the max - pooling to 2. Next, we investigated one by one the impacts of the number of convolutional kernels in the convolutional neural network, the embedding dimension of the graph attention network (for the sake of consistency, we set the embedding dimension of the convolutional neural network to be the same as that of the graph attention network), the dropout rate, and the learning rate on the model’s performance.

**FIGURE 3:**
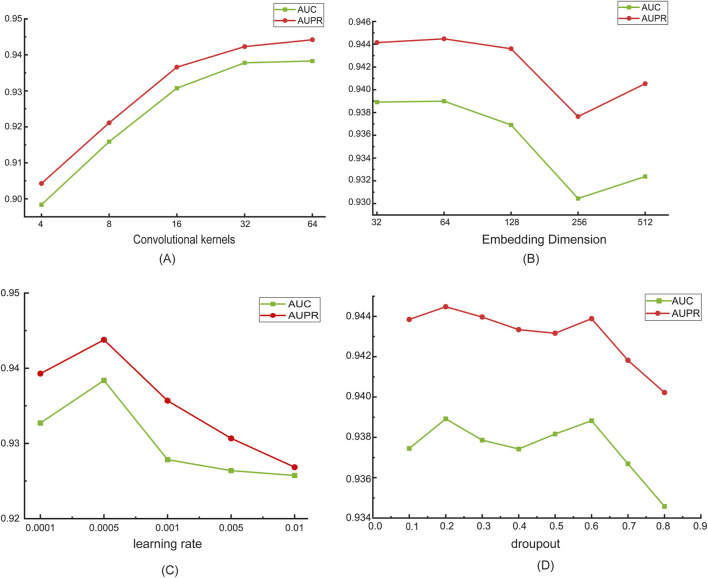
Parameter Sensitivity Analysis. **(A)** The number of convolutional kernels; **(B)** Embedding dimension; **(C)** Learning rate; **(D)** Dropout rate.

Inspired by TransRM (Liu et al., 2025), we set the number of convolutional kernels to 4, 8, 16, 32, and 64. As can be clearly observed from Figure 3A, as the number of convolutional kernels increases, the performance of the model shows a steady upward trend. It shows that the increase in the number of convolutional kernels enables the network to capture richer and more complex features. Based on this result, in this study, we defaulted the number of convolutional kernels to 64 to fully unleash the performance potential of the model.

Different embedding dimensions in the graph attention network have different impacts on the model’s performance. In order to capture the key features of the graph data while avoiding overfitting, refer to GIAE-DTI (Wang et al., 2024a), we set the dimensions to gradually increase from 32 to 512, and attempt to find the appropriate dimension that can fully describe the data features. We set the embedding dimensions as 32, 64, 128, 256 and 512. As shown in Figure 3B, as the embedding dimension increases, the AUC and AUPR values of the model first rise and then fall. When the embedding dimension is set to 64, the model achieves optimal performance. Since the secondary structure of the Nm-modified sequence is relatively simple, when the embedding dimension is too low, the model struggles to capture sufficient key information from the data. Conversely, when the embedding dimension is too high, it leads to excessive information redundancy, increasing the computational burden and potentially introducing noise interference. Therefore, we set the default value of the embedding dimension to 64 to balance model performance and information utilization efficiency.

It is evident from Figure 3C that the learning rate has a significant impact on the experimental results. Refer to DualC (Guo et al., 2025), we experimented with several different learning rate values, including 0.0001, 0.0005, 0.001, 0.005, and 0.01. The experimental results show that when the learning rate is set to 0.0005, the model exhibits optimal performance. This fully demonstrates that the learning rate, as a hyperparameter, plays a crucial regulatory role in the model training process. When the learning rate is too small, the step size of parameter updates during model training is too short, resulting in an extremely slow convergence rate. The model may require a large number of training epochs to achieve good performance, failing to fully learn the effective features in the data. On the other hand, when the learning rate is too large, the step size of parameter updates is too big, causing the model difficultly to converge, leading to poor performance. When the learning rate is set to 0.0005, the model has a moderate parameter update step size, thus achieving the best performance.

During the model training process, overfitting is a common problem, which causes the model to perform excellently on the training set but have poor generalization ability on the test set or new data. Dropout, as a simple and effective regularization technique, can significantly alleviate this problem. In this experiment, to investigate the impact of the dropout rate on model performance, refer to GIAE-DTI (Wang et al., 2024a), we set dropout rates as 0.1, 0.2, 0.3, 0.4, 0.5, 0.6, 0.7, and 0.8. Through in-depth analysis of the experimental data in Figure 3D, we found that the change in the dropout rate has a very significant impact on model performance. When the dropout rate is set too low, the model cannot effectively suppress the co-adaptation between neurons, and the overfitting problem remains severe. When the dropout rate is too high, the model randomly discards too many neurons, resulting in a large loss of useful information learned by the model and causing underfitting. When the dropout rate is set to 0.2, the model can effectively prevent overfitting while retaining enough useful information, fully learn the feature patterns of the data, and significantly improve the model’s generalization ability. Based on this, in the subsequent model training and optimization process of this study, we fixed the dropout rate at 0.2.

### 3.2 Ablation study

When predicting Nm methylation modification sites, the MultiV_Nm model integrates sequence features, chemical property features, and secondary structure features. These features reflect the characteristics of biomolecules from different dimensions. The sequence features contain the genetic information of base arrangement, the chemical property features reflect the chemical properties of molecules, and the secondary structure features describe the spatial folding morphology of molecules. Together, they provide support for accurate prediction.

To deeply analyze the specific roles of these three features in the model, a feature ablation experiment was constructed. By removing one or two features respectively, we compared the performance of the simplified model with that of the original model that fuses the three features. Table 2 clearly lists the feature combinations used in each experimental method.

**TABLE 2:** Feature combinations in the ablation study.

Method name	Sequence	Chemical property	Second structure
Seq	✓		
Che		✓	
Str			✓
Seq + Che	✓	✓	
Seq + Str	✓		✓
Che + Str		✓	✓
MultiV_Nm	✓	✓	✓

**FIGURE 4:**
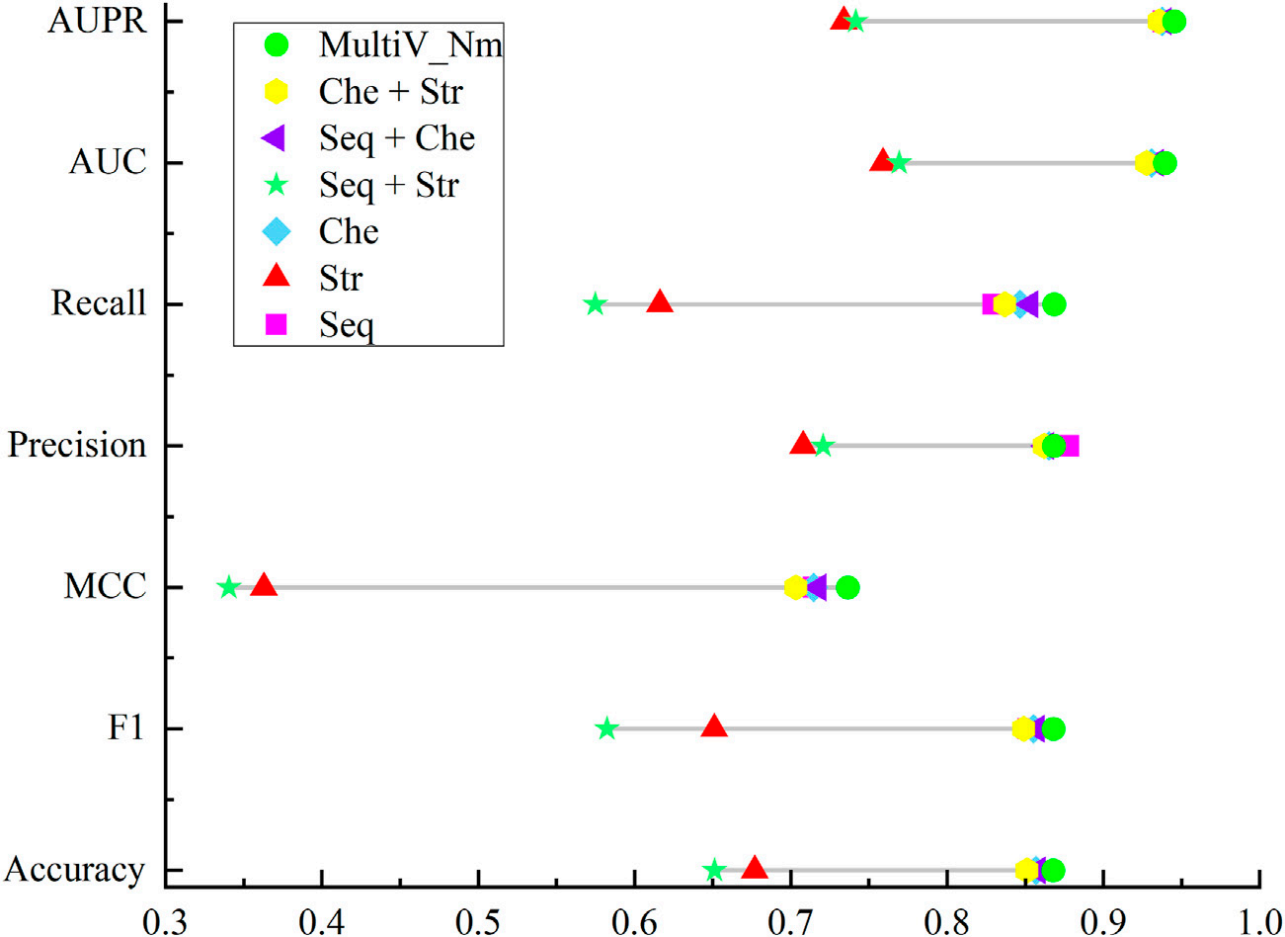
Comparison of the results of the ablation experiment.

The results of the ablation experiment are shown in Figure 4. As can be seen from Figure 4, when the three features are used separately, the prediction effects of only the sequence features and the chemical property features are not very different, while the prediction effect of using the secondary structure features is the worst. This indicates that in the scenario of predicting Nm methylation sites, sequence information and chemical properties can more effectively characterize the features of Nm methylation sites. In contrast, the secondary structure has obvious limitations. On the one hand, the secondary structure analysis focuses on the spatial conformation of RNA and cannot reflect the specific composition of the sequence, making it difficult to capture the local features in the sequence. On the other hand, when the sequence to be analyzed is too short, there will be large errors in the predicted secondary structure, resulting in the inability to provide reliable support for the prediction of Nm methylation sites. In addition, different combinations of features have different effects on the prediction of Nm methylation sites. Through research, it has been found that in the case of pairwise feature combinations, the combination of sequence features and chemical property features has the most prominent prediction effect; the combination of chemical property features and secondary structure features has a slightly inferior prediction effect. It is worth noting that the Multi_Nm model, by fusing the three features of sequence features, chemical property features, and secondary structure, has demonstrated the most excellent performance in predicting methylation sites, further improving the accuracy and reliability of the prediction.

**FIGURE 5:**
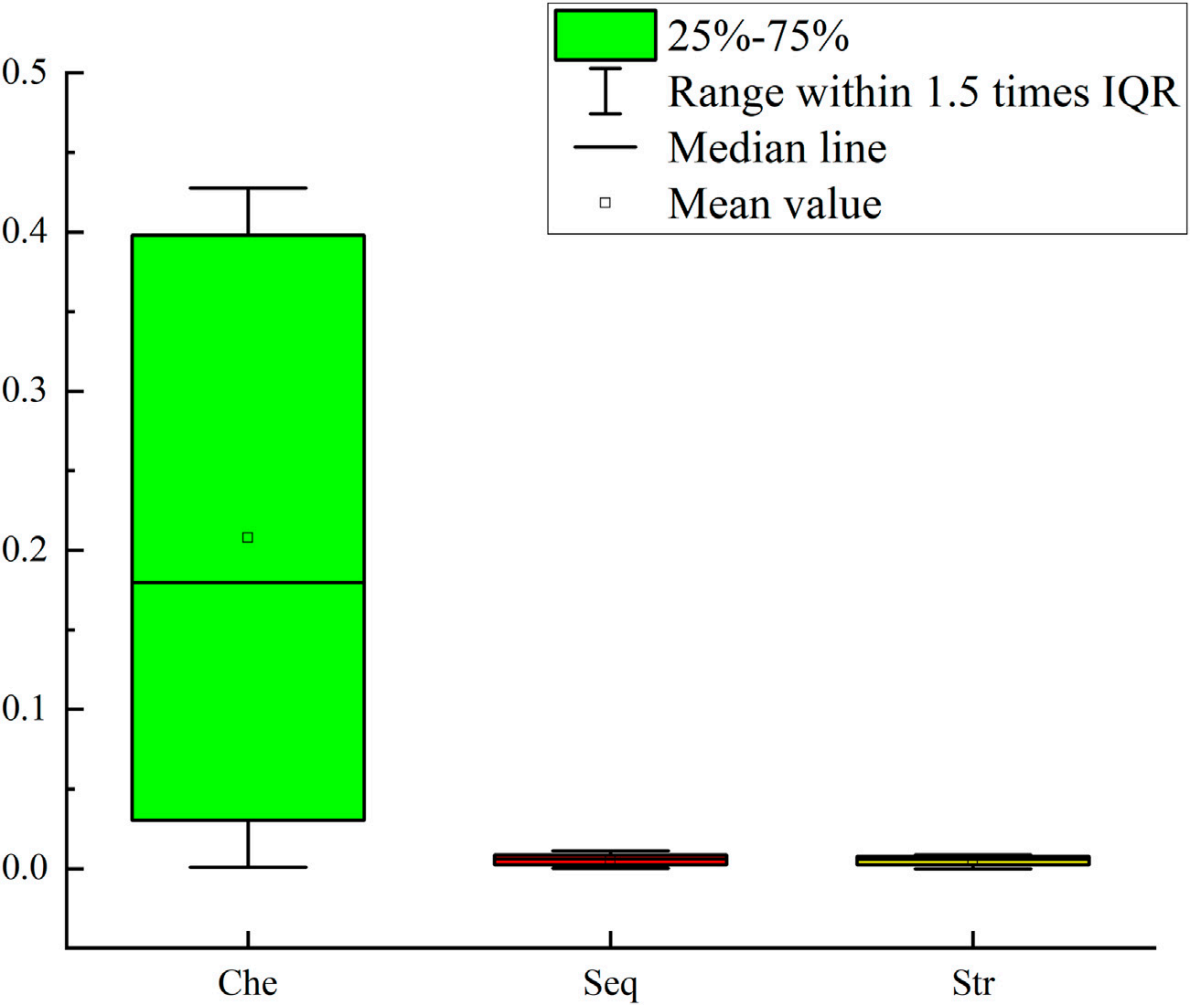
Boxplots of the importance of three types of features.

To further illustrate the importance of each feature for the model’s prediction, we evaluated the importance of the three types of features using a permutation-based feature importance calculation method. Taking the sequence features as an example, each time the order of one sequence feature was shuffled. Through five-fold cross-validation, we calculated the difference between the *AUC* of the model based on the permuted feature and the *AUC* of the standard model to obtain the importance of the sequence feature. The processing method for chemical property features and secondary structure features was the same as that for sequence features, and an importance vector with 51 dimensions was obtained for each type of feature. We plotted the importance of the three types of features using boxplots. As can be seen from Figure 5, when the chemical property features were permuted, the model was affected to the greatest extent, while the impacts of the sequence features and secondary structure features were relatively small. However, the degree of influence of the sequence features on the model was still higher than that of the secondary structure features.

### 3.3 MultiV_Nm performance under 5-CV and 10-CV

Cross-validation is an important means to evaluate the generalization ability of a model. Here, we demonstrate the performance of the MultiV_Nm model in the scenarios of five-fold cross-validation (5CV) and ten-fold cross-validation (10CV). In five-fold cross-validation, the dataset is evenly divided into five parts. One part is taken as the test set in turn, and the remaining four parts are used as the training set. The training and testing processes are repeated five times. Similarly, in ten-fold cross-validation, the dataset is divided into ten parts for operation.

**FIGURE 6:**
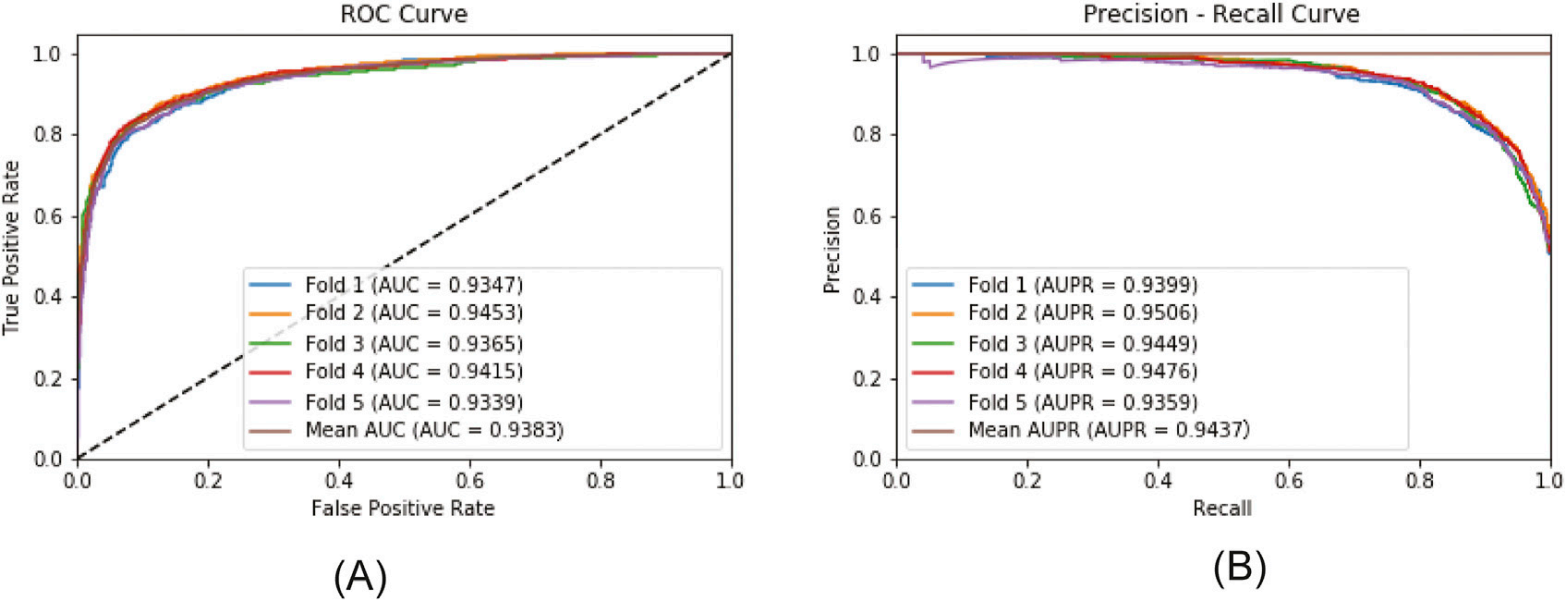
The ROC and PR curves under 5CV. **(A)** ROCcurve; **(B)** PR curve.

**FIGURE 7:**
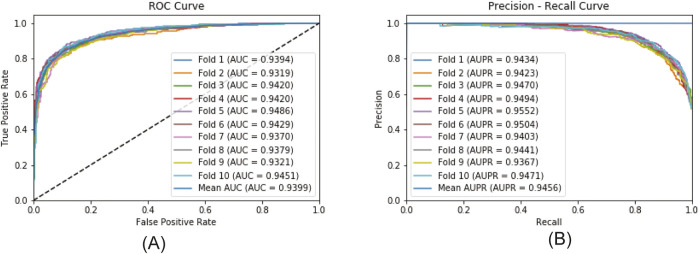
The ROC and PR curves under 10CV. **(A)** ROCcurve; **(B)** PR curve.

Figure 6 plots the ROC curve and the PR curve generated during the 5CV process. Figure 7 presents the performance of the model under 10CV. Through analysis, it is found that, whether in the case of 5CV or 10CV, the fluctuation range of the curves obtained from each fold of validation is extremely small. At the same time, the *AUC* and *AUPR* of the model both remain at a relatively high level. This fully demonstrates that the MultiV_Nm model has strong robustness and stability, and is capable of maintaining good generalization ability and prediction accuracy under different data distributions.

To further evaluate the robustness of the model, statistical methods were used for analysis. Ten rounds of 5CV and 10CV were respectively carried out, and their means and standard deviations were calculated for analysis. The specific results are shown in Table 3. As can be seen from Table 3, the results of the ten experiments are quite close to a single experiment. In the ten experiments, regardless of whether it is 5CV or 10CV, the standard deviation of each indicator is less than 0.05, while the standard deviations of *AUC* and *AUPR* are both less than 0.01. This indicates that MultiV_Nm has excellent robustness and is minimally affected by the randomness of the divided dataset.

**TABLE 3:** Comparison of the results of multiple experiments.

Cross-validation	ACC	F1_score	Precision	Recall	MCC	AUC	AUPR
5CV (single round)	0.8587	0.8582	0.8629	0.8563	0.7198	0.9383	0.9437
5CV (10 rounds)	0.8639	0.8630	0.8701	0.8583	0.7297	0.9389	0.9445
	±0.0072	±0.0079	±0.0218	±0.0284	±0.0135	±0.0045	±0.0044
10CV (single round)	0.8647	0.8641	0.8700	0.8604	0.7312	0.9399	0.9456
10CV (10 rounds)	0.8678	0.8668	0.8747	0.8608	0.7372	0.9405	0.9463
	±0.0116	±0.0116	±0.0260	±0.0289	±0.0224	±0.0071	±0.0066

### 3.4 Cross-independent testing

**FIGURE 8:**
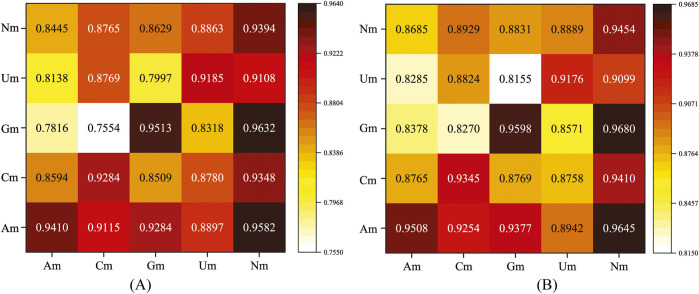
Results of AUC and AUPR in the cross-independent test. **(A)** AUC; **(B)** AUPR.

Nm methylation modifications mainly include four types, namely, Am, Cm, Gm, and Um. Next, we conducted cross-independent tests using the MultiV_Nm model. Specifically, the model was trained using four single types of Nm (i.e., Am, Cm, Gm, and Um) and the total Nm containing all types. Then, independent test sets were used respectively to evaluate the performance of the trained model. The evaluation metrics selected were the *AUC* and *AUPR*. The experimental results are shown in Figure 8.

In Figure 8, the horizontal axis represents the data types used for model training, and the vertical axis represents the data types used for testing. As can be clearly seen from the data presented in the chart, when the total Nm is used for model training, regardless of which single type of Nm is used for testing, the model can achieve relatively ideal prediction results. When a single type of Nm is used for training, the model can only achieve the optimal prediction performance when the corresponding type of Nm is used for testing.

Through in-depth analysis of these experimental results, we can infer that the total Nm contains the information of all types of Nm. This enables the model to fully learn the characteristic patterns shared by different types of Nm during the training process, thus possessing a broader adaptability and generalization ability. Therefore, when testing a single type of Nm, it can demonstrate good prediction performance. When a single type of Nm is used for training, the characteristic patterns learned by the model are highly matched to that specific type of Nm. So, when predicting the same type of Nm, due to the consistency of the characteristic patterns, the model can more accurately capture the patterns in the data, thereby obtaining better prediction results.

### 3.5 Comparison with existing methods

To comprehensively verify the effectiveness of the algorithm adopted by the MultiV_Nm model, we carried out comparative tests. The MultiV_Nm model was compared with NmRF based on machine learning and Deep-2′-O-Me based on deep learning in independent test. NmRF relies on the website http://lab.malab.cn/∼acy/NmRF to provide prediction services. When predicting Nm methylation sites, this website only outputs the prediction results and cannot provide more derivative data. To ensure the consistency and fairness of the comparative tests, we selected *Precision*, *Recall*, *ACC*, *MCC*, and *F1_score* to quantitatively evaluate the prediction performance of each model. In addition, when using Deep-2′-O-Me to test, the predicted probabilities of all sites are less than 0.5. After repeated experiments and analysis, in order to enable this method to effectively output results, we set the threshold to 0.3.

As can be seen from Table 4, the MultiV_Nm model significantly outperforms the NmRF and Deep-2′-O-Me methods in various evaluation indicators. The *ACC* of the MultiV_Nm reaches 0.8679, which has a very prominent advantage compared with 0.5419 of the NmRF and 0.6580 of the Deep-2′-O-Me. In terms of the *MCC* indicator, 0.7365 of the MultiV_Nm is much higher than 0.0953 of the NmRF and 0.3331 of the Deep-2′-O-Me. The experimental results show that the MultiV_Nm has excellent performance in the prediction of Nm methylation sites.

**TABLE 4:** Performance comparison for Nm methylation sites prediction.

Method	Accuracy	F1	MCC	Precision	Recall
NmRF	0.5419	0.6299	0.0953	0.5284	0.7797
Deep-2′-O-Me	0.6580	0.7046	0.3331	0.6201	0.8160
MultiV-Nm	0.8679	0.8681	0.7365	0.8683	0.8686

## 4 Conclusion

In the realm of RNA modification research, Nm methylation modification is pivotal. It participates in key biological processes. Precise identification of Nm methylation sites aids in uncovering disease mechanisms and developing novel diagnostic and treatment strategies. In this paper, we proposed MultiV_Nm, a multi-view feature - based prediction framework for 2′-O methylation sites. On the basis of separately extracting the sequence features, chemical features, and secondary structure features of Nm methylation sites, we used a convolutional neural network and a graph attention network, and combined them with a cross-attention mechanism to predict the Nm methylation sites. Compared with existing methods, MultiV_Nm performs excellently in multiple evaluation indicators.

However, MultiV_Nm still has some limitations. First, the model relies on high-quality RNA modification data. When extracting secondary structure features, if the data accuracy is low, it will lead to inaccurate secondary structure prediction, thereby reducing the prediction accuracy. Second, this study only used information on human Nm modification sites and did not extend it to the prediction of Nm modification sites in other species. Third, although MultiV_Nm can be extended to the prediction of other types of RNA modification sites, for different types of RNA modifications, it may be necessary to redesign the feature extraction methods and model structure to adapt to their unique modification patterns and characteristics. Even so, MultiV_Nm can still provide new ideas and insights for the prediction of RNA modification sites in different species and different types, helping to promote basic research and potentially bringing breakthroughs in the field of biomedicine.

## Data Availability

The original contributions presented in the study are included in the article/Supplementary Material, further inquiries can be directed to the corresponding authors.
